# Portable Electronic Tongue Based on Microsensors for the Analysis of Cava Wines

**DOI:** 10.3390/s16111796

**Published:** 2016-10-27

**Authors:** Pablo Giménez-Gómez, Roger Escudé-Pujol, Fina Capdevila, Anna Puig-Pujol, Cecilia Jiménez-Jorquera, Manuel Gutiérrez-Capitán

**Affiliations:** 1 Instituto de Microelectrónica de Barcelona (IMB-CNM), CSIC Campus UAB, 08193 Cerdanyola del Vallès, Spain; pablo.gimenez@csic.es (P.G.-G.); roger.escude@csic.es (R.E.-P.); cecilia.jimenez@csic.es (C.J.-J.); 2 Institut Català de la Vinya i el Vi (IRTA-INCAVI), Plaça Àgora 2, 08720 Vilafranca del Penedès, Spain; finacapdevila@gmail.com (F.C.); apuigpujol@gencat.cat (A.P.-P.)

**Keywords:** portable equipment, electrochemical microsensors, electronic tongue, multiparametric analysis, Cava wine samples

## Abstract

Cava is a quality sparkling wine produced in Spain. As a product with a designation of origin, Cava wine has to meet certain quality requirements throughout its production process; therefore, the analysis of several parameters is of great interest. In this work, a portable electronic tongue for the analysis of Cava wine is described. The system is comprised of compact and low-power-consumption electronic equipment and an array of microsensors formed by six ion-selective field effect transistors sensitive to pH, Na^+^, K^+^, Ca^2+^, Cl^−^, and CO_3_^2−^, one conductivity sensor, one redox potential sensor, and two amperometric gold microelectrodes. This system, combined with chemometric tools, has been applied to the analysis of 78 Cava wine samples. Results demonstrate that the electronic tongue is able to classify the samples according to the aging time, with a percentage of correct prediction between 80% and 96%, by using linear discriminant analysis, as well as to quantify the total acidity, pH, volumetric alcoholic degree, potassium, conductivity, glycerol, and methanol parameters, with mean relative errors between 2.3% and 6.0%, by using partial least squares regressions.

## 1. Introduction

Multiparametric analysis is a key issue for quality assurance in many different areas of interest, such as the industrial processes [1], the food industry [2], clinical diagnostics [3], or environmental monitoring [4]. In order to obtain real-time information about the composition of a sample, automatic and portable systems for decentralized analysis are highly valuable. A promising alternative is the application of electronic tongues, which generate multivariate analytical data, enlarging the number of parameters that can be determined simultaneously [5]. An electronic tongue entails the use of an array of sensors with partially-selective responses, plus a multivariate chemometric tool, and permits qualitative and/or quantitative applications in liquid media [6,7]. Among the different chemical sensors, microelectrodes fabricated with semiconductor technology present some advantages that make them particularly suitable for integration into arrays for on-site measurements, such as the miniaturization, robustness, high reproducibility, low output impedance, mass fabrication, and ease of integration with the electronic circuitry [8,9].

The applicability of electronic tongues has been especially relevant in food quality control and safety, where the increasing demand on a sustainable and high-quality production has promoted the development of more automated and precise analytical systems for monitoring [10,11]. Wine is one of the most used beverages to test the viability of these systems [12]. Cava is a quality sparkling wine protected under a designation of origin (D.O.) in Spain, which is produced mostly in the Penedès region. Unlike most wines, sparkling wines are characterized by the presence of CO_2_ in solution, which is produced by a second alcoholic fermentation, and a biological aging in contact with lees under anaerobic conditions for at least nine months in the bottle [13]. It is significant to mention the complexity of the Cava wine as a sample, given the drastic changes in the chemical composition (CO_2_, sugars, ethanol, pH, amino acids), physical properties (turbidity, density, color), and varietal aromas produced by these fermentation and aging processes [14]. Only one research group from the Universitat Autònoma de Barcelona has approached the analysis of Cava with electronic tongue systems. This group was able to classify Cava wine samples according to the content of sugar added [15] and to the aging time in bottle [16], as well as to determine the sugar and the total dry extract by using a voltammetric electronic tongue based on modified graphite-epoxy electrodes. In another work, the use of enzyme-modified sensors in the array allowed the quantification of different phenolic indices in Rosé Cava wines [17]. However, these systems were limited to the determination of just a few parameters, so it is necessary to develop more versatile and innovative tools for the analysis of Cava wine.

Most electronic tongue systems reported until now for food quality are laboratory versions [10,11], partly due to the use of large-sized sensors and data collection equipment. On one hand, the miniaturization of the electronic tongue has been approached by using individual wire electrodes [18] or developing integrated arrays of sensors. Usually, these arrays have a planar configuration and include layers of conductive inks or pastes sequentially deposited onto insulating and chemically-inert substrates. Depending on the thickness of these layers, integrated arrays of sensors have been fabricated by using screen-printed methods (thick-film technology) and applied as portable devices for monitoring drinking waters [19] and beer discrimination [20]. Additionally, thin-film technologies have been also used to fabricate integrated multisensor systems combined with flow injection analysis [9] and portable taste sensors [21], both by using standard photolithographic techniques.

On the other hand, an alternative to obtaining versatile portable instruments for multiparametric applications at a minimum cost is the use of commercial integrated circuits (IC), such as power supplies, analog-to-digital converters, and microcontrollers [22]. The reduced size of these systems implies reduced fabrication and maintenance costs, as well as lower power consumption. In a previous paper [23], we developed and tested a compact multisensor meter, whose electronics were fabricated according to microsensor requirements and took into account the minimum energy consumption and its portability. In this work, we have fabricated a multi- ion-selective field effect transistor (ISFET) meter able to simultaneously measure up to six ISFETs, with the same requirements of portability and low power consumption. The two compact meters have been used together to perform the multiparametric analysis with an array of microsensors fabricated with microelectronic technology. This combination supposes an advance to achieve a portable electronic tongue system. The array of microsensors was formed by one conductivity sensor, one redox potential (ORP) sensor, and two amperometric gold microelectrodes, which were measured with the multisensor meter, together with six ISFETs sensitive to pH, Na^+^, K^+^, Ca^2+^, Cl^−^, and CO_3_^2−^, measured with the multi-ISFET meter. For the data treatment, two different multivariate methods were used: linear discriminant analysis (LDA) and partial least squares (PLS). A set of 78 Cava wine samples was analyzed with the electronic tongue. The system demonstrated its reliability for Cava wines according to the aging time, as well as the quantification of some chemical parameters with high accuracy.

## 2. Materials and Methods 

### 2.1. Reagents and Solutions

All reagents used were of high purity, analytical grade or equivalent. All solutions were prepared with de-ionized water. For ISFET calibration, stock solutions with ionic salts with concentrations of 10^−4^, 10^−2^, and 1.0 M were prepared. In the case of those sensitive to cations (Na^+^, K^+^, and Ca^2+^), the corresponding chloride salts were considered. For the Cl^−^ and CO_3_^2−^ ions, solutions of NaCl and NaHCO_3_, respectively, were prepared. For the pH ISFET calibration, a universal buffer solution containing 0.04 M boric acid, 0.04 M acetic acid, 0.04 M phosphoric acid, and 0.1 M KNO_3_ as a background was prepared. A solution containing 0.1 M KNO_3_ was used to activate the amperometric gold electrodes. In order to calibrate the conductivity sensor, two different standard solutions from Crison (Barcelona, Spain), with nominal values of 1413 μS/cm and 147 μS/cm, were utilized. Two standard redox solutions from Panreac (Barcelona, Spain), with values of 468 mV and 220 mV (at 25 °C vs. Ag/AgCl), were used to test the ORP sensor. For the ISFET measurements, a reference solution containing an average concentration of the main species present in wine was prepared. The composition of this solution has been reported elsewhere [24].

### 2.2. Cava Wine Samples

A total set of 78 Cava wine samples from different producers were analyzed. All samples were produced and bottled in the Catalonia region and they are all commercially available. Samples were selected according to their type, taking into account their vintage time as categorized by the Regulatory Board of Cava [25]: 20 “Young” samples (9–15 months), 25 “Reserva” samples (15–30 months), and 16 “Gran Reserva” samples (more than 30 months). Moreover, a set of 17 “Rosé” Cava samples were included in this study. These samples were mainly from the Penedès region (Spain). White Cava wines were obtained mainly from Macabeu, Xarel·lo, and Parellada grape varieties, although Chardonnay and/or Subirat parent may also be used, that is, the five different white grape varieties authorized by Regulatory Board of Cava [25]. For Rosé cava wines, Trepat, Monastrell, Grenache Noir, and/or Pinot Noir might be used.

Volumetric alcoholic degree (VAD), total acidity, pH, potassium ion, conductivity, glycerol, and methanol were analyzed with reference/standard methods [26,27] in all 78 Cava samples at the Catalan Institute of Vine and Wine (IRTA-INCAVI) in order to compare and evaluate the results of the developed electronic tongue.

### 2.3. Sensors and Devices Used

A set of ISFET sensors were fabricated using standard microelectronic technology [28]. One ISFET was used for measuring pH and the rest were modified with polymeric membranes sensitive to Na^+^, K^+^, Ca^2+^, Cl^−^, and CO_3_^2−^ ions. Polymeric membranes were based on photocurable polymers with commercial ionophores from Fluka (Buchs, Switzerland). The ionophores used in each case were: 4-tert-butylcalix [4] arenetetraacetic acid tetraethyl ester (Ionophore X) for Na^+^, valinomycin (Ionophore I) for K^+^, N,N,N′,N′-tetracyclohexyl-3-oxapentanediamide (Ionophore II, ETH 129) for Ca^2+^, tridodecylmethylammonium chloride for Cl^−^, and 4-butyl-α,α,α-trifluoroacetophenone (Ionophore IV) for CO_3_^2−^. All of these ionophores are selective to the principal ion, but are not specific and they present a certain degree of cross-response to other ions in solution. Membrane composition and preparation has been presented elsewhere [29,30,31,32]. An Orion 90-02-00 double junction Ag/AgCl reference electrode (Thermo Electron, Waltham, MA, USA) with 0.1 M CH_3_COOLi solution in its outer chamber was employed for all of the potentiometric measurements.

Sensors based on a platinum four-electrode configuration were employed as the conductivity sensors and ORP sensors. Their fabrication and characterization are reported elsewhere [33]. Finally, two conventional thin-film gold electrodes, also fabricated according to standard photolithographic techniques, were employed to perform chronoamperometric measurements. The amperometric cell contained the working electrode, a platinum electrode as a counter electrode (Radiometer, Lyon, France), and an Ag/AgCl/10% (w/v) KNO_3_ reference electrode (Metrohm 0726 100, Herisau, Switzerland).

All of these microsensors present a long-term stability above seven months with discrete calibrations in aqueous solutions [29,30,31,32,33]. However, their lifetimes are, in fact, limited by their use, so that in a continuous monitoring application, for example in Cava wine production, their response characteristics (sensitivity, selectivity) would degrade within 2–3 months. This effect is noticed especially in ISFET sensors due to the leaching of the ionophores out of the polymeric membrane [29].

### 2.4. Measurement Equipment

The measurements with the conductivity sensor, the ORP sensor, and the two amperometric gold electrodes were performed with a multisensor meter constructed on the IMB-CNM premises. A detailed explanation of the electronic design, software for data collection, and global performance of this equipment is presented in [23].

For the measurements with the six ISFETs, a new electronic device was fabricated. The electronic board was designed with Allegro PCB Designer and Layout Plus software (Cadence Design Systems, Bracknell, UK). The fabrication of the board was molded using a PhotoMap s43 milling machine (LPKF Laser and Electronics AG, Garbsen, Germany). The size of the system is 21 cm × 10 cm × 3 cm. The PCB was formed by four different areas: the power supply unit, digital part, analog part, and six ISFET connectors. The communication between the digital and analog parts was performed using the I2C protocol. In order to obtain a real-time simultaneous measurement of the six ISFETs, each channel had its own I2C address. The ISFET measurement was carried out by applying a 100 µA current between the drain and the source, and recording the ISFET gate potential (in mV). This potential is related with the analyte concentration in solution.

The digital interface permitted the establishment of communication between the user and the analog electronic part. The main IC was the ADUC848BSZ62-5 microcontroller (μC) (Analog Devices, Norwood, MA, USA). This μC was composed by a central processing unit (CPU), memory, digital and analog ports, and units for standard communication protocols. One of the memories (E2PROM) contained the programmed code, which was sequentially executed by the μC. This code was programmed in the C++ language using the development kit μVision 4.02 (Keil Electronik, Grasbrunn, Germany). The visualization of the results and the configuration of the measurement parameters were carried out employing a virtual instrument (VI). The VI was programmed with LabView 2013 (National Instruments, Austin, TX, USA). This is a modular, versatile, quick, and intuitive software program which provides a clear working environment to the user.

**Figure 1:**
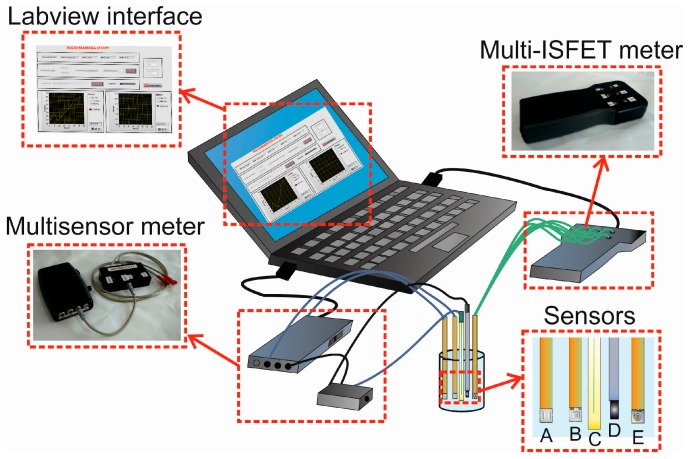
Scheme of the whole measurement system. In detail: (**A**) conductivity or ORP sensor; (**B**) amperometric sensor; (**C**) reference electrode; (**D**) counter electrode; and (**E**) ISFET.

A scheme of the whole system formed by the two portable meters, a laptop PC with the Labview software, and the measurement cell with the different microsensors is shown in Figure 1.

### 2.5. Characterization of Sensors and Electronics

For the evaluation of the new multi-ISFET meter, the response characteristics of ISFETs sensitive to the different ions considered were studied. The response characteristics were evaluated by calibration in response to the principal ion. These calibration curves were obtained by means of the method of the analyte addition: the variation of potential originated by the addition of accumulated microvolumes of stock ion solutions (10^−4^, 10^−2^, and 1.0 M) in 25 mL of de-ionized water was measured and registered by the new equipment. In the case of the pH ISFET, microvolumes of 1 M NaOH solution were added to a 50 mL of universal buffer solution to change pH from 2 to 12. Then, the potential (in mV) was plotted versus the logarithm of the activity of the principal ion (log a_x_ or pH), where the sensitivity, limit of detection, or linear range of each potentiometric sensor were extracted. All of these experiments were performed at room temperature by using three different ISFETs of each type, prepared under the same experimental conditions.

Moreover, two platinum four-electrode sensors and two thin-film gold electrodes were firstly chemically cleaned, followed by an electrochemical activation carried out in 0.1 M KNO_3_ where the electrode was cycled from +0.8 V to −2.2 V at least 20 times. These sensors were also characterized before the analysis using the multisensor meter. The response characteristics are reported in [23], including the conductivity calibration and the ORP test obtained with the four-electrode sensors.

### 2.6. Electronic Tongue Measurement Procedure

The analysis was directly carried out in the Cava sample, previously degassed by magnetic stirring. No measurement replications were done in order to get a rapid analysis and prevent changes of the Cava wine, as well as to minimize the formation of CO_2_ bubbles onto the sensor surface.

Once calibrated, the six ISFETs (one for each ion considered) were immersed in the Cava wine and the potentials (in mV vs. Ag/AgCl) were recorded every 10 s for 30 s using the multi-ISFET meter. The output values corresponding to the relative measurements of each ISFET with respect to the reference solution, which was checked periodically, were used as analytical signals for the models. This is a common strategy to correct the possible drift of the ISFET sensors.

Once the good behavior of the two platinum four-electrode sensors and two thin-film gold electrodes was confirmed, they were immersed in the Cava wine sample and the signals were recorded every 1 s for 30 s using the multisensor meter. In the case of the amperometric measurements, one gold electrode was set to an overpotential of +1.01 V (vs. Ag/AgCl), at which the polyphenols are probably oxidized. The other gold electrode was set to +1.31 V (vs. Ag/AgCl), related with the oxidation of gold from the electrode [2].

The measurements with these two devices were performed sequentially, first with the multi-ISFET meter and then with the multisensor meter. Therefore, a complete analysis of one Cava wine sample took around 2 min under batch conditions.

### 2.7. Data Treatment and Analysis

Once all of the samples were passed through the sensors, a data matrix was constructed with the different variables to be used as the input of the chemometric tools. In this study, the input data were composed by 10 variables, as shown in Table 1.

These data were treated using different multivariate methods. The linear discriminant analysis (LDA) was utilized to achieve a good classification model for the Cava wine samples. Discriminant analysis is a supervised method, since it is used to build linear combinations of the original variables for a number of pre-specified classes (model). These combinations are later used for allocating new and unknown samples to the most probable class. Another important application of discriminant analysis is to help in interpreting differences between groups of samples.

The partial least squares (PLS) regression was employed to perform the quantification of different parameters of the samples. PLS is a method for multivariate calibration that finds the combinations of the original variables (components or factors) that will best predict the values of the parameters analyzed, by maximizing the covariance between the matrices. In this work, the PLS-1 variant (one PLS model per each parameter) was used in order to obtain more accurate predictions.

For the two methods, the original values were previously autoscaled—all of the variables were centered and set to a standard deviation equal to 1, to avoid variables from having a different influence on the model. Additionally, all of the obtained models were centered. On one hand, the Mahalanobis method, for measuring the distance of an observation to the centers of the groups, together with the leave-one-out cross validation method were used for the LDA model. On the other hand, the classical non-linear iterative (NIPALS) algorithm, together with the test-set validation technique, was used for the PLS regressions. In this case, a fixed calibration set composed of 60 Cava wine samples was chosen. Meanwhile, the prediction set consisted of 18 samples. To control all of these parameters and to perform the analyses, the Unscrambler v.9.1 informatics package (CAMO ASA, Oslo, Norway) was used.

## 3. Results and Discussion

### 3.1. Characterization of Sensors and Electronics

ISFET sensors selective to pH, Na^+^, K^+^, Ca^2+^, Cl^−^, and CO_3_^2−^ were calibrated with the new device. Three sensors of each type were connected and measured simultaneously with the multi-ISFET meter. The response characteristics obtained are shown in Table 2. The limit of detection is calculated by the cross-point method recommended by IUPAC for potentiometry [34]. As can be observed, all of the sensors presented a Nerstian response for at least a two-decade linear range with a high significant regression coefficient (R^2^). In fact, these analytical parameters obtained with the new device are very similar to those reported previously for ISFETs with the same membrane composition [24,29,30,31], also in terms of the limit of detection. These results demonstrate the good performance of the developed equipment. Simultaneous measurements of six ISFETs can be carried out without any electrical interference thanks to the circuit design, which is a key issue for an electronic tongue system. Therefore, this multi-ISFET meter was used to perform the analysis of the Cava wine samples.

### 3.2. Classification of the Cava Wine Samples

With the data obtained from the different variables, LDA was performed. The confusion matrix of the obtained model is presented in Table 3, together with the percentages of sensitivity and specificity for the four groups. The sensitivity corresponds to the samples of each group correctly classified by the LDA model, while the specificity is calculated as the samples of different groups correctly rejected by the model. As shown in Table 3, especially good results are obtained in the prediction of the Reserva class samples, with just one sample confused with the Gran Reserva class. Looking more in detail at the specificity of classification, it is observed that no Young sample is confused with Gran Reserva, and, conversely, no Gran Reserva sample is confused with Young. This is because the system is able to discriminate very different aging times that are 9–15 months (Young) and more than 30 months (Gran Reserva). On the other hand, the Reserva class has an aging time in between (15–30 months) and, therefore, it is more likely to overlap with the borderline samples. However, the values of specificity are above 90% in all cases and the total sensitivity of prediction is 87%. It is also important to highlight the high percentage of sensitivity and specificity achieved for the Rosé class samples, which demonstrates the great discrimination capacity of the system formed by just electrochemical microsensors.

### 3.3. Quantification of Legal Parameters

Next, a PLS regression was realized in order to assess if the system was able to quantify some chemical parameters of the samples already analyzed with standard methods. These parameters are of interest to meet the legal limits, such as the total acidity, pH, and VAD. These legal limits are fixed between 10.8% and 12.8% for VAD, between 2.8 and 3.3 for pH, and at least 5 g/L for total acidity [35]. For the regression, the prediction set was formed by 18 Cava wine samples, whose data was not included in the calibration process: four Young (Y 3336, 3709, 4956, 5219), six Reserva (R 2719, 2929, 3727, 5241, 5608, and 5962), four Gran Reserva (GR 2720, 3182, 4183, and 5220), and four Rosé (Ro 2978, 3103, 4814, and 5017). The results are shown in Table 4. As can be seen, the interpolated values are in good agreement with the data obtained using standard methods for the three parameters. In general, the relative errors are below 9%. Especially good results are obtained for pH and VAD prediction, with relative errors below 5%. The results for VAD are significant since there is no specific sensor for this parameter. Values for total acidity are also quite accurate considering that it is a global parameter that includes all titratable acids, mainly tartaric acid, but also lactic acid, malic acid, citric acid, etc., and again no specific sensor is used.

### 3.4. Quantification of Other Parameters

PLS regressions were also used to determine other parameters of interest, such as potassium and conductivity, which are related with the tartaric stabilization, and glycerol and methanol, both related with the final sensory quality of the Cava wine. The data for the prediction set obtained with our system and with standard methods are shown in Table 5. The values obtained with both methods are also in good agreement, with relative errors below 15%. As can be seen, the mean errors are below 6.0% for the four parameters. The best results are obtained for conductivity prediction. Again, glycerol and methanol are determined with no specific sensors, with good accuracy.

## 4. Conclusions

A compact electronic tongue has been developed and applied to measure Cava wines. The electronics used (multi-ISFET meter and multisensor meter) have been fabricated according to microsensor requirements and taking into account the minimum energy consumption. This power consumption is below 10 mA if we consider the measurement with the 10 microsensors simultaneously every 15 min. This means that the equipment could work continuously up to 150 h using a standard 9 V battery.

This electronic tongue has been applied to the analysis of 78 Cava wine samples. The qualitative results confirm that the device is capable of classifying the samples according to the aging time and to distinguish the Rosé samples from the white Cava wine samples with a high sensitivity and specificity. In addition, the application of the PLS technique to the collected data permits the quantification of some chemical parameters of interest in the final product. Some of these parameters have legal limits to accomplish, such as the total acidity, pH, and VAD. In general, the relative errors are below 10%. The best results are obtained for pH, VAD, and conductivity predictions with mean relative errors below 4%. The good accuracy obtained for the determination of VAD, glycerol, and methanol is especially relevant since there are no specific sensors for these parameters.

Compared with other electronic tongues for Cava wine analysis [15,16,17], this system is able to determine simultaneously up to seven important chemical parameters, apart from the qualitative analysis, thanks to the hybrid nature of the electrochemical sensors (potentiometric, amperometric, and conductimetric). Moreover, this e-tongue uses small and low-power equipment for measurement, instead of bench-top laboratory equipment, and microsensors, which are easy to miniaturize and integrate with the electronics.

In conclusion, the good results obtained both for classification and quantification analyses confirm the viability of the multiparametric system. Additionally, the use of portable meters together with electrochemical microsensors fabricated with semiconductor technology provide an advantageous combination for rapid and feasible in-field measurements, not only for the wine industry but for food quality control in general.

## Figures and Tables

**Table 1:** Variables considered for constructing the models.

Equipment	Sensors	Variables
Multi-ISFET meter	Six ISFETs	pH, Na^+^, K^+^, Ca^2+^, Cl^−^, and CO_3_^2−^
Multisensor meter	Two four-electrode sensors	Conductivity and ORP
	Two gold electrodes	Current at +1.01 V and +1.31 V

**Table 2:** Response characteristics obtained from the calibration curves for each type of ISFET using the multi-ISFET meter.

Parameter	Sensitivity (mV/dec) ^1^	Linear Range (M)	R^2^	Limit of Detection (M)
pH	54.2 (0.5)	pH 1.56–11.42	0.9998 (n = 10)	-
Na^+^	54.0 (0.4)	2.1 × 10^−5^–2.2 × 10^−2^	0.9994 (n = 6)	5.9 × 10^−6^
K^+^	57.0 (0.8)	2.0 × 10^−5^–2.1 × 10^−2^	0.9996 (n = 7)	1.8 × 10^−6^
Ca^2+^	28.6 (0.6)	5.9 × 10^−7^–1.9 × 10^−2^	0.9998 (n = 9)	1.9 × 10^−7^
Cl^−^	−59 (1)	2.0 × 10^−4^–2.1 × 10^−2^	0.9998 (n = 4)	2.8 × 10^−5^
CO_3_^2−^	−58 (2)	2.1 × 10^−4^–2.2 × 10^−2^	0.9993 (n = 4)	3.0 × 10^−5^

^1^ Standard deviation of three different ISFET is indicated in brackets.

**Table 3:** Confusion matrix for the Cava wine samples obtained with the LDA model using the cross-validation method.

Classes	Prediction				Sensitivity (%)	Specificity (%)
	Young	Reserva	Gran Reserva	Rosé		
Young	16	2	0	2	80	97
Reserva	0	24	1	0	96	91
Gran Reserva	0	3	13	0	81	98
Rosé	2	0	0	15	88	97

**Table 4:** Results of legal parameter quantification with the electronic tongue using PLS-1 regression. Standard method data were provided by INCAVI.

Sample ^1^	Total Acidity (g/L)			pH			VAD (%)		
	Standard Method	Electronic Tongue	Relative Error (%)	Standard Method	Electronic Tongue	Relative Error (%)	Standard Method	Electronic Tongue	Relative Error (%)
Y 3336	6.5	5.9	8.5	3.04	3.23	6.1	11.75	11.97	1.8
Y 3709	6.1	6.0	1.3	3.01	3.17	5.4	12.15	11.97	1.5
Y 4956	6.5	6.1	6.4	2.94	2.95	0.3	11.85	11.87	0.2
Y 5219	6.7	6.2	8.1	3.03	3.07	1.3	11.55	11.82	2.4
R 2719	5.5	5.8	5.0	3.31	3.35	1.2	13.00	12.06	7.2
R 2929	6.1	6.1	0.4	3.07	3.08	0.4	12.30	11.86	3.5
R 3727	6.1	6.2	0.9	2.96	3.04	2.8	11.75	11.90	1.3
R 5241	5.8	6.4	11.0	3.43	3.44	0.4	12.05	11.76	2.4
R 5608	6.2	6.1	2.4	2.94	3.15	7.3	12.15	11.92	1.9
R 5962	5.6	6.1	9.0	3.08	3.08	0.1	12.10	11.89	1.7
GR 2720	5.5	5.7	4.4	3.15	3.05	3.2	12.85	12.05	6.2
GR 3182	5.5	6.1	11.6	3.02	3.00	0.6	12.15	11.91	2.0
GR 4183	5.5	5.6	1.3	3.02	3.04	0.7	12.40	12.07	2.7
GR 5220	6.5	7.0	7.4	2.93	3.10	5.9	11.75	12.01	2.2
Ro 2978	5.5	5.9	7.8	3.35	3.19	4.9	12.20	12.15	0.4
Ro 3103	6.2	6.7	7.8	3.01	3.04	1.0	12.20	12.11	0.7
Ro 4814	5.8	6.0	3.2	3.00	3.10	3.4	12.30	11.96	2.8
Ro 5017	5.5	6.1	11.3	3.02	3.05	1.1	12.05	11.99	0.5
	**Mean Relative Error**		**6.0**	**Mean Relative Error**		**2.6**	**Mean Relative Error**		**2.3**

**^1^** Y: Young; R: Reserva; GR: Gran Reserva; Ro: Rosé.

**Table 5:** Results of quality parameter quantification with the electronic tongue using PLS-1. Standard method data were provided by INCAVI.

Sample ^1^	Potassium (mg/L)			Conductivity (mS/cm)			Glycerol (g/L)			Methanol (mg/L)		
	Standard Method	Electronic Tongue	Relative Error (%)	Standard Method	Electronic Tongue	Relative Error (%)	Standard Method	Electronic Tongue	Relative Error (%)	Standard Method	Electronic Tongue	Relative Error (%)
Y 3336	355	360	1.3	1.34	1.33	0.9	5	5.5	9.7	29	30	3.8
Y 3709	433	423	2.4	1.42	1.32	6.9	4.9	5.4	10.0	29	28	2.3
Y 4956	326	328	0.6	1.29	1.26	2.1	-	-	-	30	30	1.2
Y 5219	332	332	0.0	1.38	1.32	4.3	5.3	5.2	1.7	29	30	5.0
R 2719	550	487	11.4	1.79	1.66	7.5	6.1	6.2	2.2	31	34	10.6
R 2929	360	395	9.7	1.29	1.28	0.9	5.1	5.7	12.2	30	32	5.7
R 3727	300	332	10.7	1.21	1.21	0.3	5.6	5.4	2.8	30	30	0.0
R 5241	372	315	15.4	1.19	1.13	5.3	5.1	5.6	9.5	30	30	1.3
R 5608	379	418	10.3	1.31	1.31	0.1	5.8	5.8	0.7	29	31	6.1
R 5962	320	334	4.4	1.2	1.19	0.5	5.5	5.7	2.9	28	30	7.5
GR 2720	544	518	4.7	1.73	1.70	1.6	6.9	6.3	8.1	31	32	3.0
GR 3182	321	327	1.9	1.14	1.16	2.0	5.1	5.4	6.1	30	30	1.5
GR 4183	391	408	4.2	1.42	1.41	0.6	5.5	5.4	2.5	28	31	10.8
GR 5220	300	301	0.2	1.33	1.29	2.9	5.1	5.3	4.4	29	28	2.3
Ro 2978	430	452	5.1	1.29	1.41	9.1	5.4	5.9	8.5	38	33	13.4
Ro 3103	367	346	5.6	1.44	1.31	9.4	5.4	5.7	4.9	38	36	5.5
Ro 4814	459	398	13.4	1.39	1.33	4.0	5.5	5.8	5.8	36	32	12.0
Ro 5017	318	306	3.6	1.22	1.20	1.7	5.4	5.7	6.2	27	30	9.7
	**Mean Relative Error**		**5.8**	**Mean Relative Error**		**3.3**	**Mean Relative Error**		**5.8**	**Mean Relative Error**		**5.6**

**^1^** Y: Young; R: Reserva; GR: Gran Reserva; Ro: Rosé.
